# Recombination in the wheat stem rust pathogen mediated by an indigenous barberry species in Spain

**DOI:** 10.3389/fpls.2023.1322406

**Published:** 2024-01-15

**Authors:** Julian Rodriguez-Algaba, Dolors Villegas, Carlos Cantero-Martínez, Mehran Patpour, Anna Berlin, Mogens S. Hovmøller, Yue Jin, Annemarie F. Justesen

**Affiliations:** ^1^ Department of Agroecology, Faculty of Science and Technology, Aarhus University, Slagelse, Denmark; ^2^ Sustainable Field Crops, IRTA, Institute of Agrifood Research and Technology, Lleida, Spain; ^3^ Agrotecnio Center, Universitat de Lleida, Lleida, Spain; ^4^ Department of Forest Mycology and Plant Pathology, Swedish University of Agricultural Sciences, Uppsala, Sweden; ^5^ USDA-ARS Cereal Disease Laboratory, University of Minnesota, St Paul, MN, United States

**Keywords:** *Puccinia graminis*, alternate host, sexual reproduction, *Berberis*, genetic diversity

## Abstract

The comeback of wheat stem rust in Europe, caused by *Puccinia graminis* f. sp. *tritici*, and the prevalence of the alternate (sexual) host in local areas have recently regained attention as a potential threat to European wheat production. The aim of this study was to investigate a potential epidemiological link between the aecia found on an indigenous barberry species and stem rust infections on nearby cereals and grasses. Aecial infections collected from *Berberis vulgaris* subsp. *seroi* were inoculated on a panel of susceptible genotypes of major cereal crop species. In total, 67 stem rust progeny isolates were recovered from wheat (51), barley (7), and rye (9), but none from oat, indicating the potential of barberry derived isolates to infect multiple cereals. Molecular genotyping of the progeny isolates and 20 cereal and grass stem rust samples collected at the same locations and year, revealed a clear genetic relatedness between the progeny isolated from barberry and the stem rust infections found on nearby cereal and grass hosts. Analysis of Molecular Variance indicated that variation between the stem rust populations accounted for only 1%. A Principal Components Analysis using the 62 detected multilocus genotypes also demonstrated a low degree of genetic variation among isolates belonging to the two stem rust populations. Lastly, pairwise comparisons based on fixation index (Fst), Nei’s genetic distances and number of effective migrants (Nm) revealed low genetic differentiation and high genetic exchange between the two populations. Our results demonstrated a direct epidemiological link and functionality of an indigenous barberry species as the sexual host of *P*. *graminis* in Spain, a factor that should be considered when designing future strategies to prevent stem rust in Europe and beyond.

## Introduction

Wheat stem rust, caused by *Puccinia graminis* f. sp. *tritici* (*Pgt*), is considered a destructive fungal disease causing major yield losses globally (Singh et al., 2016; Patpour et al., 2022). *Pgt* is a heteroecious rust fungus requiring two unrelated host plant species to complete its life cycle. Asexual reproduction occurs on the cereal host, whereby urediniospores with long distance dispersal capacity can cause polycyclic infections in the same growing season. Sexual reproduction takes place on an alternate host plant species of *Berberis* and *Mahonia* genera (Stubbs, 1985). Besides *Pgt*, other special forms of *P. graminis* adapted to certain cereals, e.g., *P. graminis* f. sp. *secalis* adapted to rye (*Pgs*) and *P. graminis* f. sp. *avenae* adapted to oat, in addition to other *Puccinia* species infecting cereals and grasses, e.g., *P. striiformis sensu lato* and *P. brachipodii s.l*., complete the sexual cycle on the alternate host (Cummins and Greene, 1966; Anikster, 1984; Naef et al., 2002; Jin et al., 2010; Rodriguez-Algaba et al., 2020; Rodriguez-Algaba et al., 2021). Stem rust has the ability to survive between crop seasons in plant debris by the production of overwintering teliospores. Under optimal environmental conditions, teliospores germinate and produce basidiospores that can infect the alternate host. Subsequently, pycnia bearing pycniospores and receptive hyphae are formed where fertilization between different mating types occurs. After fertilization, aecia containing aeciospores are formed leading to the generation of novel genetic diversity and virulence combinations which may pose a detrimental effect to the durability of wheat rust resistance (Buller, 1950; Leonard and Szabo, 2005).

More than 100 *Berberis* and *Mahonia* species have been confirmed susceptible to *Pgt* (Ahrendt, 1961; Roelfs, 1985). The occurrence of *Berberis* species in Europe, and particularly *B. vulgaris* (aka European or common barberry), has significantly increased following the repeal of eradication laws during the 20^th^ century and more recently by its reintroduction by conservation groups (Berlin et al., 2013; Saunders et al., 2019). Barberry eradication in major wheat growing areas in Europe and North America and breeding efforts for wheat rust resistance have prevented significant stem rust epidemic outbreaks during the last decades (Stakman, 1923; Peterson et al., 2005). Recently, stem rust has emerged in Europe with multiple outbreaks reported on bread and durum wheat (Bhattacharya, 2017; Firpo et al., 2017; Patpour et al., 2022). Additionally, high genetic and virulence diversity have been detected in *Pgt* isolates collected in areas associated with the presence of *Berberis* spp. in Europe, which imply that sexual reproduction may play a role in the epidemiology of stem rust in these areas (Patpour et al., 2022).

The role of the alternate host in wheat stem rust epidemiology is mostly based on studies of *B. vulgaris*. In addition to *B. vulgaris*, two additional barberry subspecies are present in Spain, i.e., *B. vulgaris* subsp*. seroi* (syn. *B. garciae*) and *B. vulgaris* subsp*. australis* (syn. *B. hispanica*) (López González, 1986). Previous studies have investigated the functionality of *B. vulgaris* and indigenous barberry species in Spain as alternate hosts of *P. graminis* by host infection studies and molecular genotyping using either the aecial structures formed on the alternate host or cereal and grass stem rust samples collected in nearby barberry areas (Patpour et al., 2022; Rodriguez-Algaba et al., 2022; Villegas et al., 2022). The objective of this study was to investigate a potential epidemiological link between the aecia found on *B. vulgaris* subsp*. seroi* and a subset of previously characterized *Pgt* isolates derived from cereal and grass stem rust samples collected in the same areas in Spain (Patpour et al., 2022). Particularly, we investigated the genetic diversity, structure, and differentiation of *Pg* progeny isolates derived from aecial infections and *Pg* uredinial infections from cereals and grasses collected in proximity to these, which allowed us to infer on the potential capacity of *Pg* of undergoing sexual reproduction and subsequently infecting adjacent wheat crops. The novel results of this study indicated that the sexual life cycle of the cereal stem rust pathogen is active in Spain, including the special form infecting wheat. This underlines the potential risk that the sexual cycle of *Pgt* may pose to wheat production in Europe and highlights the need to reinitiate stem rust resistance breeding strategies to achieve durable rust resistance in wheat.

## Materials and methods

### Sample collection

**Figure 1:**
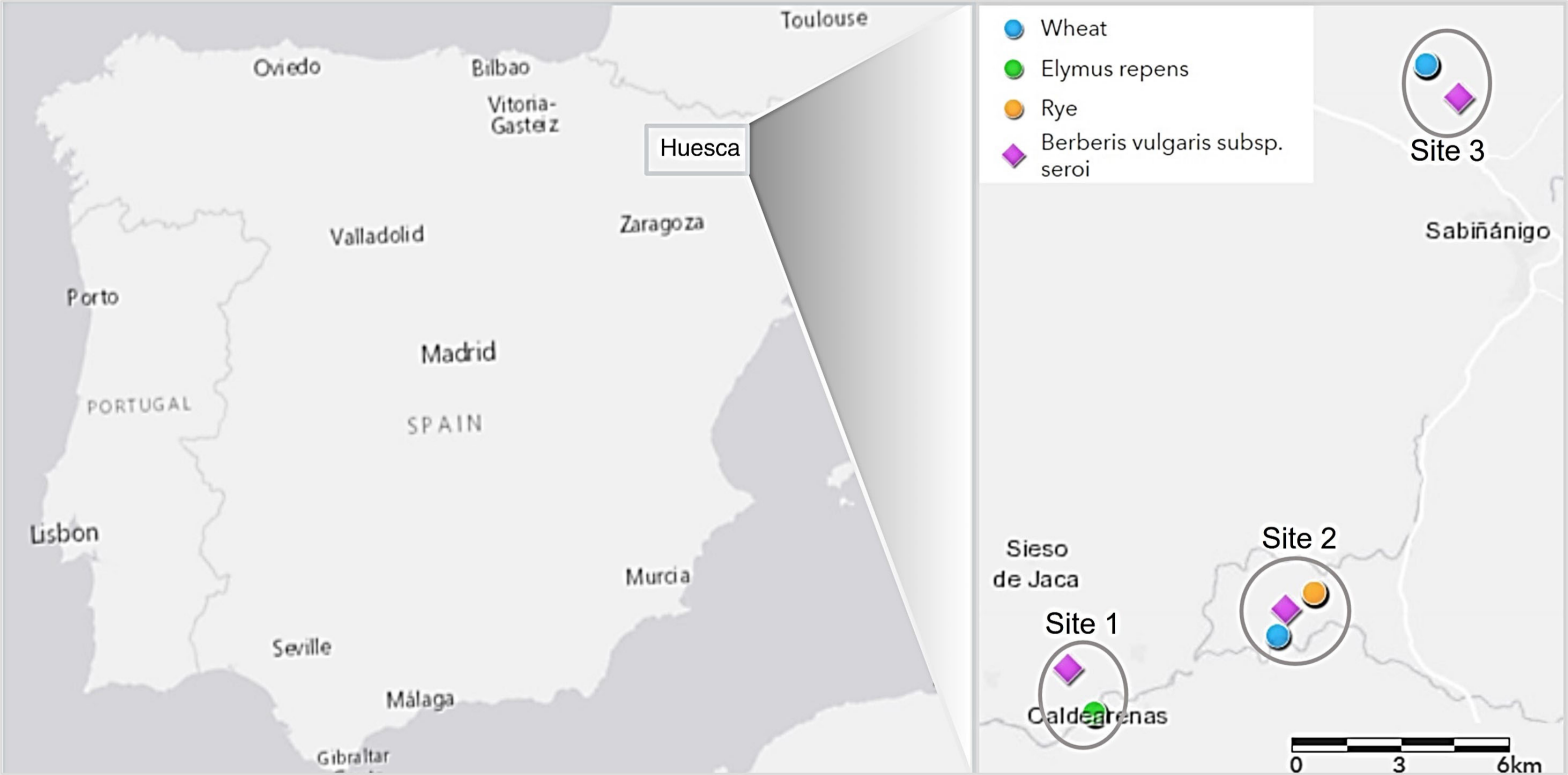
Geographical location of barberry and cereal and grass stem rust samples collected at three different sampling sites in Huesca province in Spain (samples from cereals and grasses previously characterized in Patpour et al. (2022) (see **Supplementary Table S1** for detailed sampling information).

Leaves of *B. vulgaris* subsp. *seroi* bearing aecia were collected from three locations in Huesca province in Spain in 2019 (**Figure 1**). Barberry subspecies identification was confirmed based on the color of one year old stems and matured fruits, and the morphology of leaves according to López González (1986). Infected barberry leaves, usually containing multiple aecial clusters, were used to investigate host specificities on major cereal crops. Subsequently, the sexually-derived *Pg* samples (progeny population) and 20 cereal/grass stem rust samples (cereal and grass population) previously characterized in Patpour et al. (2022) and randomly collected in close proximity to the same locations and year as for the infected barberry leaves were used to investigate the functionality of *B. vulgaris* subsp. *seroi* as an alternate host for *Pgt* in Spain under natural conditions (**Figure 1**; **Supplementary Table S1**).

### Cereal host specificities

Host specificities of stem rust samples were investigated by pooled aecia inoculations on susceptible varieties of wheat (*Triticum aestivum* var. Morocco and Line E), rye (*Secale cereale* var. Prolific), barley (*Hordeum vulgaris* var. Hiproly), and oat (*Avena sativa* var. Marvelous) (Rodriguez-Algaba et al., 2022; Villegas et al., 2022). To investigate the capability of aecia to infect cereals, the procedures described in Rodriguez-Algaba et al. (2022) were followed. Two pots of each cereal variety were randomly placed below infected barberry leaves and incubated at 15°C for 24h in darkness, 100% relative humidity (RH). Subsequently, seedlings were transferred to spore-proof greenhouse cabins. Stem rust pustules were observed from approximately 10 days after aeciospore exposure, after which leaf segments bearing stem rust pustules were detached and dried at room temperature for later use.

### Molecular genotyping

DNA extraction and molecular genotyping using 19 microsatellite markers was carried out following the methods described in Patpour et al. (2022). DNA was extracted using the stem rust lesions sampled from the cereal varieties of wheat, barley and rye. Allele sizes of the 20 cereal and grass stem rust samples were directly extracted from Patpour et al. (2022).

### Population genetic analyses

Genetic analyses of uredinial progeny isolates recovered from wheat, barley, and rye and the 20 stem rust isolates sampled from cereals and grasses were carried out using the “poppr” package version 2.9.3 implemented in the R environment (Kamvar et al., 2014; Kamvar et al., 2015; R Core Team, 2022). The “mlg.filter” strain was used to collapse multilocus genotypes (MLGs) with missing values that shared alleles with other MLGs without considering missing values. Population genetic parameters included number of MLGs, genotypic richness measured by the number of MLGs divided by number of samples and genetic diversity measured by the Simpson’s index (lambda) (Simpson, 1949). Expected (He) and observed (Ho) heterozygosity, and inbreeding coefficients (Fis = (Mean He - Mean Ho)/Mean He) were calculated in GenAlEx 6.503 with 9999 permutations and bootstraps (Peakall and Smouse, 2006; Peakall and Smouse, 2012). Analysis of molecular variance (AMOVA) was performed on clone-corrected data to assess molecular variations among populations and host of origin (Excoffier et al., 1992). The fixation index (Fst) measuring divergence between populations, Nei´s genetic distance and the number of effective migrants (Nm = [(1/Fst)-1]/4) were calculated using GenAlEx 6.503 with 9999 permutations and bootstraps (Wright, 1965; Nei, 1972; Peakall and Smouse, 2006; Peakall and Smouse, 2012). Principal Component Analysis (PCA) was performed to infer in the genetic variability of uredinial isolates derived from the two stem rust populations using the “ade4” package version 1.7.20 implemented in the R environment (Dray and Dufour, 2007; Bougeard and Dray, 2018).

## Results

### Cereal host specificities

**Figure 2:**
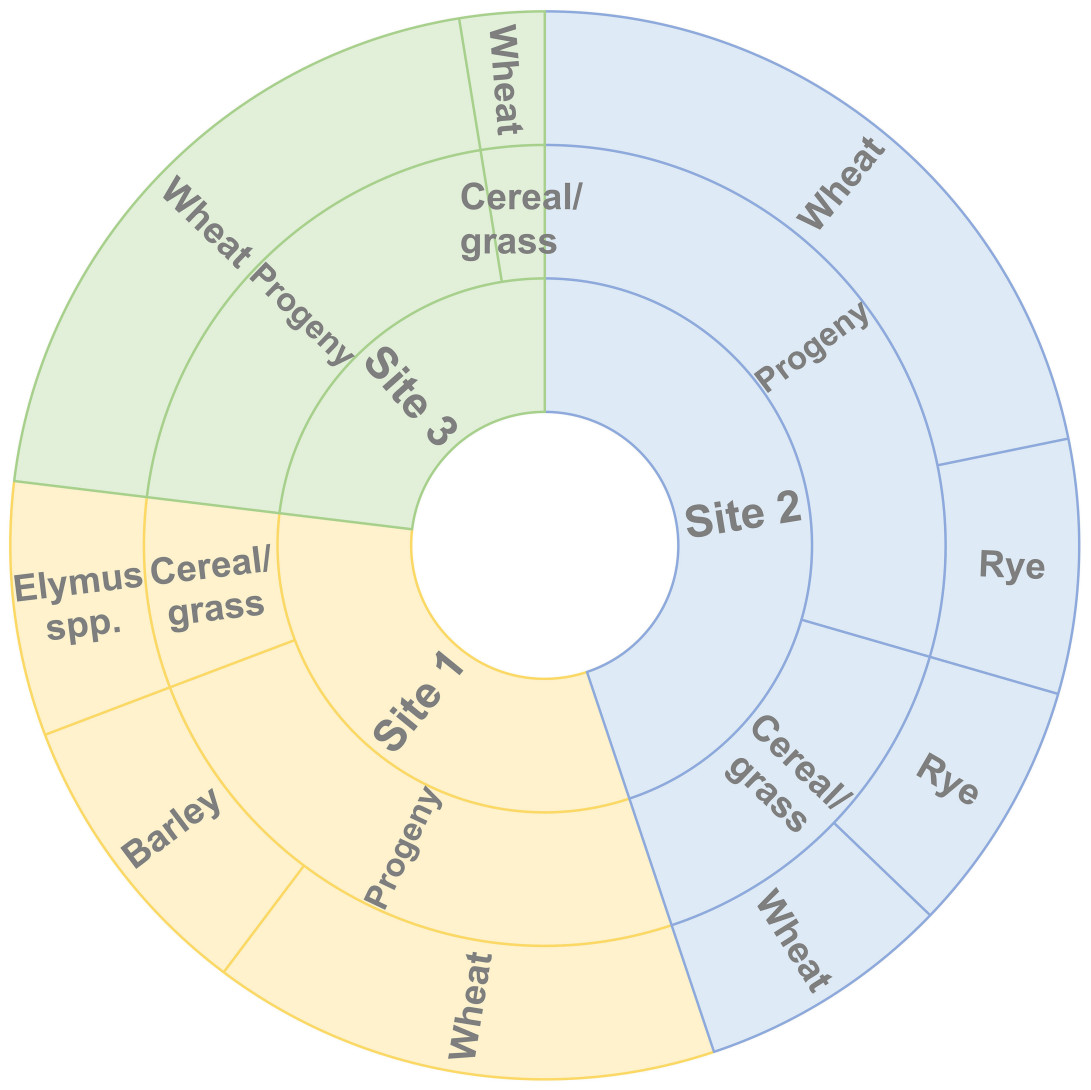
Host specificity (outer circle) of samples collected at the three sampling sites (inner circle) in Huesca province (Spain) derived from the aecial progeny and the cereal and grass stem rust populations (middle circle).

Aecial samples collected from *B. vulgaris* subsp. *seroi* at three neighboring sampling sites resulted in the recovery of 67 stem rust pustules from varieties of wheat, barley, and rye, respectively (**Table 1**). The number of recovered isolates varied across cereal varieties and sampling sites and no pustules were observed on oat. This indicated the presence of *P. graminis* with specificities for wheat and rye, i.e., *Pgt* and *Pgs*. The distribution of samples collected at the three sampling sites derived from both the aecial progeny and the cereal and grass stem rust populations according to host of origin is shown in **Figure 2**. This indicated that samples from wheat (12), barley (7) and *Elymus* spp (6). were recovered from site 1, samples from wheat (23) and rye (12) from site 2 and samples from wheat (18) from site 3.

**Table 1:** Number of single lesions of *Puccina graminis* derived from barberry leaves bearing aecia sampled in the Huesca province in Spain and recovered on multiple varieties of major cereal crops.

Population	Cereal crops					
	Wheat		Barley	Rye	Oat	
	Line E	Morocco	Hiproly	Prolific	Marvelous	Total
Progeny	20	31	7	9	0	67

### Genetic diversity and molecular variance

**Figure 3:**
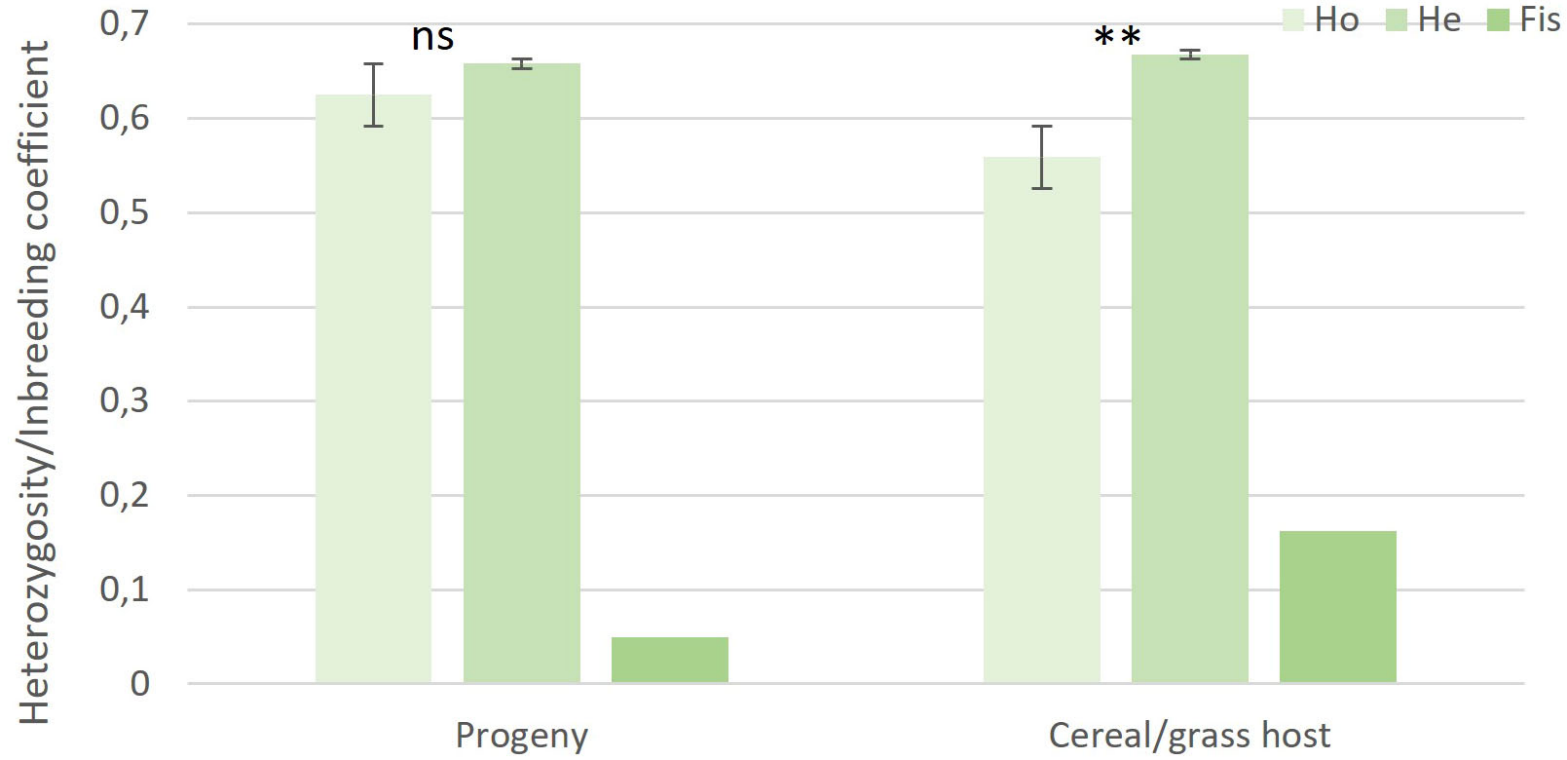
Observed heterozygosity (Ho), expected heterozygosity (He) and inbreeding coefficients (Fis) of aecial progeny and cereal and grass populations of *Puccinia graminis*. Vertical bars represent standard errors (SE), level of significance: ***P < 0.001, **P < 0.01, * P < 0.05, ns, not significant.

The genotype accumulation curve confirmed the suitability of the 19 SSR markers for capturing the genetic variation within the genotyped samples (**Supplementary Figure S1**). Out of the 67 recovered isolates, 58 isolates were successfully genotyped. Population genetic analysis performed on the 58 progeny isolates and the 20 cereal and grass stem rust sample population from the surrounding area (Patpour et al., 2022) revealed 62 MLGs (**Table 2**; **Supplementary Table S2**). The allele sizes for each of the 78 stem rust isolates, their host of origin and associated MLG are provided in **Supplementary Table S2**. MLG62, recovered from the aecial progeny population, was resampled from rye and wheat, respectively. The high number of MLGs identified, high values of genotypic richness (0.76-0.90) and genotypic diversity (0.98-0.94), and lower observed than expected heterozygosity were all strong signatures of sexual reproduction (**Table 2**). Fis values close to zero indicated a certain degree of inbreeding in both populations (**Figure 3**).

**Table 2:** Genetic diversity parameters of aecial-derived progeny isolates of *Puccinia graminis* (current study) and isolates of *P. graminis* collected from cereal and grasses (Patpour et al., 2022) at 19 microsatellite loci.

Population	N^b^	MLGs^c^	Genotypic richness^d^	Genotypic diversity^e^
Progeny	58	44	0.759	0.977
Cereal/grass host	20	18	0.900	0.944
Total^a^	78	62	0.795	0.984

a Indices calculated for pooled populations.

b Number of genotyped samples.

c Number of multilocus genotypes.

d Number of MLGs divided by number of genotyped samples.

e Simpson’s genotypic diversity index.

The AMOVA results indicated significant variations in the two variables analyzed, i.e., population and host (**Table 3**). The highest variation was observed within samples for the two variables (84%–85%). Molecular variation among samples within populations and hosts accounted for 14% and 13%, respectively. Variation among populations and hosts accounted for only 1%. Similarly, variation between populations and among hosts accounted for only 1% and 2%, respectively.

**Table 3:** Analysis of molecular variance (AMOVA) of *Puccinia graminis* isolates for two individual hierarchies, i.e., population and host.

Source of variation	Df^a^	Sum of squares	Mean of squares	Estimated variance	Variation (%)	*p*-value^b^
Population and Host						
Between populations	1	24.58	24.58	0.09	1	0.094
Among hosts within populations	4	73.09	18.27	0.21	2	0.038
Among samples within hosts	57	855.02	15.00	1.78	13	<0.001
Within samples	63	721.05	11.45	11.45	84	<0.001
Population						
Between populations	1	24.67	24.67	0.19	1	0.007
Among samples within populations	60	910.16	15.17	1.85	14	<0.001
Within samples	62	711.63	11.48	11.48	85	<0.001
Host						
Among Hosts	3	59.86	19.95	0.21	2	0.013
Among samples within hosts	59	892.83	15.13	1.84	13	<0.001
Within samples	63	721.05	11.45	11.45	85	<0.001

^a^Degrees of freedom, ^b^p-values based on 1999 permutations.

### Genetic structure and differentiation

**Figure 4:**
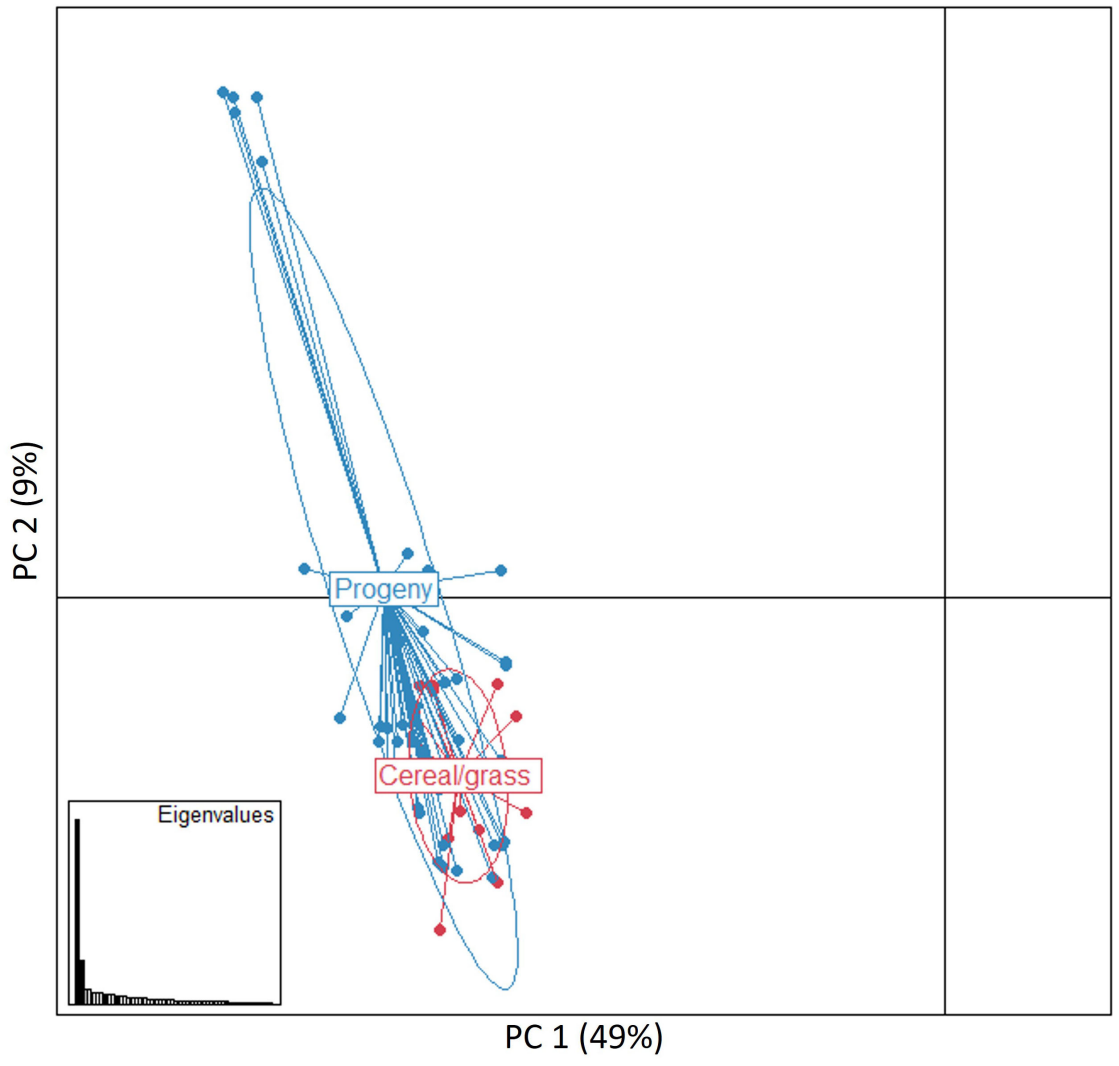
Principal component (PC) analysis for the 62 multilocus genotypes (MLGs) detected in the progeny and cereal and grass stem rust populations. The PC1 and PC2 axes explained 49% and 9% of the genetic differences detected between individual samples, respectively. Each dot represents a distinct multilocus genotype. Ellipses indicate the distribution of samples from the two stem rust populations. Eigenvalues indicate the amount of genetic information retained by the PC analysis (bottom left inset).

A PCA analysis conducted using all 62 MLGs revealed that isolates belonging to the progeny and the cereal and grass stem rust populations highly intersect, indicating a low genetic variation among most isolates (**Figure 4**). Isolates from the two the stem rust progeny and cereal and grass populations did not cluster in regard to collection site or host of origin. The first two principal components explained almost 60% of the genetic variance observed between individual samples, i.e., PC1 (49%) and PC2 (9%) (**Figure 2**). Pairwise comparisons based on Fst values, Nei’s genetic distances and number of effective migrants (Nm) indicated a low genetic differentiation between the two stem rust populations. This was exemplified by significant low values of Fst (0.034, p < 0.001) and Nei´s genetic distance (0.130), and high values of Nm (7.018).

## Discussion

Recent reports about the comeback of wheat stem rust coupled with the increasing occurrence of the sexual host have regained attention to this almost forgotten disease as *Berberis* spp. may have a profound effect in the epidemiology of stem rust and a potential threat to cereal production in Europe (Saunders et al., 2019; Patpour et al., 2022; Rodriguez-Algaba et al., 2022). In the present study, we provided evidence of the completion of the sexual life cycle of the cereal stem rust pathogen on an indigenous barberry species in Spain and subsequent infection on the cereal host under natural conditions. We used a population genetic approach to analyze sexual progeny derived from aecial clusters collected from *B. vulgaris* f. sp. *seroi* and cereal and grass stem rust samples collected in the same areas. This allowed us to investigate the epidemiological link between *Pg* isolates originated from the aecial structures on barberry and from nearby cereal and grass hosts, respectively. The results provided novel insights about the functionality of barberry spp. as sexual hosts of *P. graminis* in Spain and unequivocally demonstrated the potential threat that the sexual host may pose in the generation of novel genetic variability and virulence combinations.

Sexual reproduction leads to the generation of novel gene and virulence combinations that could pose a threat to cereal production (Roelfs and Groth, 1980). The role of the alternate host in the epidemiology of wheat stem rust in Europe has historically attracted the attention of plant pathologists, which has nowadays been emphasized due to the repeal of eradication laws during the last century (Stakman, 1923; Hermansen, 1968; Berlin et al., 2012). In fact, high genetic diversity and unusual virulence combinations associated with the alternate host was previously reported in Europe and beyond for stem rust samples infecting wheat, rye, and oat (Roelfs and Groth, 1980; Berlin et al., 2012; Berlin et al., 2013; Berlin et al., 2014; Olivera et al., 2019). Recently, the role of the alternate host in the epidemiology of stem rust infecting cereals and grasses in Europe has been reported using phylogenetic and *in vivo* analyses of aecia collected from multiple barberry species (Lewis et al., 2018; Rodriguez-Algaba et al., 2022; Villegas et al., 2022). Furthermore, the functionality of barberry species in Spain has recently been investigated using cereal and grass stem rust samples collected near the alternate host (Olivera et al., 2022; Patpour et al., 2022). In these studies, *P. graminis* samples collected from wheat, rye, and barley carrying unusual virulence combinations to widely deployed resistance genes such as *Sr31* and *Sr59* were detected in indigenous barberry areas in Spain. This has led to the assumption that the sexual cycle may be involved in the generation of these novel virulence combinations. Stem rust variability has previously been hypothesized to be associated with the presence of *Berberis* spp. in proximity to wheat fields in Spain (Stakman, 1923). Further studies have reported on the sexual capacity of aecial structures collected in Spain to generate pathogen races carrying unusual virulence combinations compared to stem rust samples collected in wheat areas without the presence of the alternate host (Urríes and Cañamas, 1952; Salazar and Brañas, 1973). However, to date, no studies have provided definitive proof of the epidemiological link between the aecial structures found on the alternate host, *Berberis* spp., and the stem rust infections found on nearby wheat crops under natural conditions in Spain.

In this study, *in vivo* assays using pooled aecial samples from *B. vulgaris* f. sp. *seroi* resulted in stem rust infections in selected varieties of wheat, barley, and rye. This probably indicated the presence of *Pgt* and *Pgs*, which is in line with recently published results from aecia collected from several barberry species in Europe (Rodriguez-Algaba et al., 2022; Villegas et al., 2022). Low genetic differentiation has been reported between these ff. spp., which may indicate some degree of overlap in cereal and grass host range (Rodriguez-Algaba et al., 2022). The stem rust infections observed on barley suggest that these were likely caused from *Pgt* or *Pgs* infections or from sexual crosses between these two special forms of *Pg* (Anikster, 1984). Interestingly, Patpour et al. (2022) reported that stem rust isolates collected from wheat, rye and *Elymus* spp. in the proximity of barberry areas in Spain were recovered on selected susceptible wheat varieties and subsequently virulence phenotyped using a differential set consisting of multiple wheat lines. Overall, this indicated a wide plasticity of *Pgt* on infecting multiple hosts including rye, barley, and wild grasses, which underlines a high ability of *Pgt* to adapt to multiple genotypes of various hosts species. Moreover, high genetic diversity with respects to number of MLGs and diversity indexes, and lower observed than expected heterozygosity were observed for both stem rust populations, which was expected for pathogen populations of sexual origin. This is in line with the results previously published in Patpour et al. (2022) using cereal and grass stem rust samples isolated from both cereals and grasses collected in the same areas in Spain. Furthermore, population genetic analysis revealed an epidemiological link between the sexually derived progeny and the stem rust infections found on the cereal and grass hosts. This conclusion was supported by AMOVA analyses, which indicated that only 1% of the overall variation could be attributed to differences between the aecial progeny and the cereal and grass stem rust populations. Additionally, the PCA revealed that isolates belonging to the two stem rust populations highly intersected indicating a low genetic variability among isolates. Lastly, pairwise comparisons based on Fst values, Nei’s genetic distances and number of effective migrants (Nm) between the aecial progeny and cereal and grass stem rust populations indicated low genetic differentiation and high genetic exchange. Overall, the low genetic differentiation observed between the two stem rust populations, which were both collected in close proximity, suggests that both populations originated from common and/or genetically similar parental ancestors.

The successful completion of the sexual life cycle of the wheat stem rust pathogen is dependent on multiple factors. For instance, the coexistence of barberry species near wheat fields is a prerequisite as basidiospores derived from germinating teliospores formed in the cereal host are not capable of travelling long distances (Roelfs and Bushnell, 1985; Zhao et al., 2016). Moreover, a synchronization between both the sexual and the cereal hosts with regards to host physiology and susceptibility is key for a successful infection on the sexual host (Zhao et al., 2016; Rodriguez-Algaba et al., 2021). In the present study, field surveys in the Huesca province in Spain revealed the presence of *B. vulgaris* subsp. *seroi* concurrently growing in the proximity of cereals and volunteer grasses. Indeed, plenty of aecia were observed in May with subsequent stem rust infections found on cereals and grasses. These observations suggested that aeciospores originating from the alternate host may serve as a primary source of infection of wheat and other cereals and grasses in Spain.

Here, we demonstrated an epidemiological link between the aecial structures formed on an indigenous barberry species and subsequent stem rust infections in cereal and grass hosts in a northern region in Spain. These results stress the importance of indigenous barberry species as a significant component of stem rust epidemiology in areas where barberry species and cereal hosts coexist. This underlines the need to continue searching in areas where conducive physiological and environmental conditions may be present for completion of the sexual life cycle of stem rust and subsequent infection of cereal hosts. The re-emergence of wheat stem rust in Spain and the occurrence of unique virulence combinations emphasize the need to continue surveying and monitoring this destructive wheat fungal disease.

## Data availability statement

The original contributions presented in the study are included in the article/**Supplementary Material**. Further inquiries can be directed to the corresponding author.

## Author contributions

JR-A: Conceptualization, Data curation, Methodology, Validation, Writing – original draft, Writing – review & editing. DV: Methodology, Writing – review & editing. CC-M: Methodology, Writing – review & editing. MP: Data curation, Writing – review & editing. AB: Writing – review & editing. MH: Conceptualization, Writing – review & editing. YJ: Conceptualization, Writing – review & editing. AJ: Conceptualization, Writing – review & editing.

## Supplementary material

Supplementary Figure 1 Genotype accumulation curve with the number of loci (n-1, maximum number of loci) and the 62 discriminated MLGs confirming the suitability of the 19 SSR markers applied in the present study.

## References

[B1] Ahrendt L. W. A. (1961). *Berberis* and *Mahonia.* A taxonomic revision. J. Linn. Soc London Bot. 369, 1–410. doi: 10.1111/j.1095-8339.1961.tb00889.x

[B2] Anikster Y. (1984). “The *formae speciales* ,” in The cereal rusts. Eds. Bushnell W. R. Roelfs A. P. (Orlando, FL: Academic Press).

[B3] Berlin A. Djurle A. Samils B. Yuen J. (2012). Genetic variation in *Puccinia graminis* Collected from Oats, Rye, and Barberry. Phytopathol. 102, 1006–1012. doi: 10.1094/PHYTO-03-12-0041-R 22734559

[B4] Berlin A. Rahmatov M. Muminjanov H. Yuen J. (2014). Sexual reproduction contributes to genotypic variation in the population of *Puccinia graminis* in Tajikistan. Eur. J. Plant Pathol. 141, 159–168. doi: 10.1007/s10658-014-0534-2

[B5] Berlin A. Samils B. Djurle A. Wirsen H. Szabo L. Yuen J. (2013). Disease development and genotypic diversity of *Puccinia graminis* f. sp. *avenae* in Swedish oat fields. Plant Pathol. 62, 32–40. doi: 10.1111/j.1365-3059.2012.02609.x

[B6] Bhattacharya S. (2017). Deadly new wheat disease threatens Europe’s crops. Nature 542, 145–146. doi: 10.1038/nature.2017.21424 28179687

[B7] Bougeard S. Dray S. (2018). Supervised multiblock analysis in R with the ade4 package. J. Stat. Software 86, 1–17. doi: 10.18637/jss.v086.i01

[B8] Buller A. H. R. (1950). Researches on fungi, vol. VII (Toronto, Canada: The University of Toronto Press).

[B9] Cummins B. Greene H. C. (1966). A review of the grass rust fungi that have uredial paraphyses and aecia on *Berberis mahonia* . Mycol. 58, 702–721. doi: 10.1080/00275514.1966.12018364

[B10] Dray S. Dufour A.-B. (2007). The ade4 package: implementing the duality diagram for ecologists. J. Stat. Software 22, 1–20. doi: 10.18637/jss.v022.i04

[B11] Excoffier L. Smouse P. E. Quattro J. M. (1992). Analysis of molecular variance inferred from metric distances among DNA haplotypes: application to human mitochondrial-DNA restriction data. Genet. 131, 479–491. doi: 10.1093/genetics/131.2.479 PMC1205020 1644282

[B12] Firpo P. D. O. Newcomb M. Flath K. Sommerfeldt-Impe N. Szabo L. J. Carter M.. (2017). Characterization of Puccinia graminis f. sp tritici isolates derived from an unusual wheat stem rust outbreak in Germany in 2013. Plant Pathol. 66, 1258–1266. doi: 10.1111/ppa.12674

[B13] Hermansen J. E. (1968). Studies on the spread and survival of cereal rust and mildew diseases in Denmark (Copenhagen, Denmark: Doctor of Science thesis, The Royal Veterinary and Agricultural University).

[B14] Jin Y. Szabo L. J. Carson M. (2010). Century-old mystery of *Puccinia striiformis* life history solved with the identification of *Berberis* as an alternate host. Phytopathol. 100, 432–435. doi: 10.1094/PHYTO-100-5-0432 20373963

[B15] Kamvar Z. N. Brooks J. C. Grünwald N. J. (2015). Novel R tools for analysis of genome-wide population genetic data with emphasis on clonality. Front. Genet. 6, 208. doi: 10.3389/fgene.2015.00208 26113860 PMC4462096

[B16] Kamvar Z. N. Tabima J. F. Grunwald N. J. (2014). Poppr: an R package for genetic analysis of populations with clonal, partially clonal, and/or sexual reproduction. PeerJ 2, e281. doi: 10.7717/peerj.281 24688859 PMC3961149

[B17] Leonard K. J. Szabo L. J. (2005). Stem rust of small grains and grasses caused by Puccinia graminis. Mol. Plant Pathol. 6, 99–111. doi: 10.1111/j.1364-3703.2005.00273.x 20565642

[B18] Lewis C. M. Persoons A. Bebber D. P. Kigathi R. N. Maintz J. Findlay K.. (2018). Potential for re-emergence of wheat stem rust in the United Kingdom. Commun. Biol. 1, 13. doi: 10.1038/s42003-018-0013-y 30271900 PMC6053080

[B19] López González G. (1986). “Berberidaceae,” in Flora iberica. Ed. López González G. (Madrid, Spain: Real Jardín Botánico, CSIC).

[B20] Naef A. Roy B. A. Kaiser R. Honegger R. (2002). Insect-mediated reproduction of systemic infections by Puccinia arrhenatheri on Berberis vulgaris. New Phytol. 154, 717–730. doi: 10.1046/j.1469-8137.2002.00406.x 33873461

[B21] Nei M. (1972). Genetic distance between populations. Am. Nat. 106, 283–292. doi: 10.1086/282771

[B22] Olivera P. D. Sikharulidze Z. Dumbadze R. Szabo L. J. Newcomb M. Natsarishvili K.. (2019). Presence of a Sexual Population of *Puccinia graminis* f. sp. *tritici* in Georgia Provides a Hotspot for Genotypic and Phenotypic Diversity. Phytopathol. 109, 2152–2160. doi: 10.1094/PHYTO-06-19-0186-R 31339468

[B23] Olivera P. D. Villegas D. Cantero-Martinez C. Szabo L. J. Rouse M. N. Luster D. G.. (2022). A unique race of the wheat stem rust pathogen with virulence on Sr31 identified in Spain and reaction of wheat and durum cultivars to this race. Plant Pathol. 71, 873–889. doi: 10.1111/ppa.13530

[B24] Patpour M. Hovmøller M. S. Rodriguez-Algaba J. Randazzo B. Villegas D. Shamanin V. P.. (2022). Wheat stem rust back in Europe: diversity, prevalence and impact on host resistance. Front. Plant Sci. 13. doi: 10.3389/fpls.2022.882440 PMC9202592 35720526

[B25] Peakall R. Smouse P. E. (2012). GenAlEx 6.5: genetic analysis in Excel. Population genetic software for teaching and research-an update. Bioinformatics 28, 2537–2539. doi: 10.1093/bioinformatics/bts460 22820204 PMC3463245

[B26] Peakall R. O. D. Smouse P. E. (2006). genalex 6: genetic analysis in Excel. Population genetic software for teaching and research. Mol. Ecol. Not. 6, 288–295. doi: 10.1111/j.1471-8286.2005.01155.x PMC3463245 22820204

[B27] Peterson P. D. Leonard K. J. Roelfs A. P. Sutton T. B. (2005). Effect of barberry eradication on changes in populations of *Puccinia graminis* in Minnesota. Plant Dis. 89, 935–940. doi: 10.1094/PD-89-0935 30786626

[B28] R Core Team. (2022). A language and environment for statistical computing (Vienna, Austria: R Foundation for Statistical Computing).

[B29] Rodriguez-Algaba J. Hovmøller M. S. Justesen A. F. (2020). Sexual recombination within the “Kranich” race of the yellow rust fungus *Puccinia striiformis* f.sp. *tritici* on *Berberis vulgaris* . Europ. J. Plant Pathol. 156, 1169–1173. doi: 10.1007/s10658-019-01919-4

[B30] Rodriguez-Algaba J. Hovmoller M. S. Schulz P. Hansen J. G. Lezaun J. A. Joaquim J.. (2022). Stem rust on barberry species in Europe: Host specificities and genetic diversity. Front. Genet. 13. doi: 10.3389/fgene.2022.988031 PMC9554944 36246643

[B31] Rodriguez-Algaba J. Hovmøller M. S. Villegas D. Cantero-Martínez C. Jin Y. Justesen A. F. (2021). Two indigenous *Berberis* species from Spain were confirmed as alternate hosts of the yellow rust fungus *Puccinia striiformis* f. sp. *tritici* . Plant Dis. 105, 2281–2285. doi: 10.1094/PDIS-02-21-0269-SC 33630692

[B32] Roelfs A. P. (1985). Wheat and rye stem rust The Cereal Rusts Vol. II: Diseases, Distribution, Epidemiology, and Control. Eds. Roelfs A. P. Bushnell W. R. (Orlando, FL: Academic Press).

[B33] Roelfs A. P. Bushnell W. R. (1985). The cereal rusts vol. II: diseases, distribution, epidemiology, and control (Orlando: Academic Press).

[B34] Roelfs A. P. Groth V. J. (1980). A comparison of virulence phenotypes in wheat stem rust populations reproducing sexually and asexually. Phytopathol. 70, 855–862. doi: 10.1094/Phyto-70-855

[B35] Salazar J. Brañas M. (1973). Physiologic races of wheat black rust (Puccinia graminis Pers. var. tritici Eriks. et Henn.) detected in Spain in the years 1968–1971 (in Spanish). Cereal Rusts Bull. 1, 21–23.

[B36] Saunders D. G. O. Pretorius Z. A. Hovmøller M. S. (2019). Tackling the re-emergence of wheat stem rust in Western Europe. Commun. Biol. 2, 51. doi: 10.1038/s42003-019-0294-9 30729187 PMC6361993

[B37] Simpson E. H. (1949). Measurement of diversity. Nature 163, 163–688. doi: 10.1038/163688a0

[B38] Singh R. P. Singh P. K. Rutkoski J. Hodson D. P. He X. Jorgensen L. N.. (2016). Disease impact on wheat yield potential and prospects of genetic control. Annu. Rev. Phytopathol. 54, 303–322. doi: 10.1146/annurev-phyto-080615-095835 27296137

[B39] Stakman E. C. (1923). Barberry eradication prevents black rust in Western Europe (USA: United States Department of Agriculture), 1–15.

[B40] Stubbs R. W. (1985). The cereal rusts. Eds. Roelfs A. P. Bushnell W. R. (Orlando, FL: Academic Press).

[B41] Urríes M. J. Cañamas R. (1952). Physiological races of "Puccinia graminis tritici" and "P. rubigo-vera tritici” in Spain, in the period 1950–1952 (in Spanish). Boletín del Instituto Nacional Investigaciones Agronómicas 27, 593–616.

[B42] Villegas D. Bartaula B. Cantero-Martínez C. Luster D. Szabo L. Olivera P.. (2022). Barberry plays an active role as an alternate host of *Puccinia graminis* in Spain. Plant Pathol 71, 1174–1884. doi: 10.1111/ppa.13540 PMC9311844 35915821

[B43] Wright S. (1965). The interpretation of population structure by F-statistics with special regard to systems of mating. Evol. 19, 395–420. doi: 10.2307/2406450

[B44] Zhao J. Wang M. Chen X. Kang Z. (2016). Role of alternate hosts in epidemiology and pathogen variation of cereal rusts. Annu. Rev. Phytopathol. 54, 207–228. doi: 10.1146/annurev-phyto-080615-095851 27296143

