# Supplementary figures and images for: Recombination in the wheat stem rust pathogen mediated by an indigenous barberry species in Spain
Source: Front Plant Sci. 2024 Jan 15;14:1322406. doi: 10.3389/fpls.2023.1322406 (PMC10825791; doi:10.3389/fpls.2023.1322406)

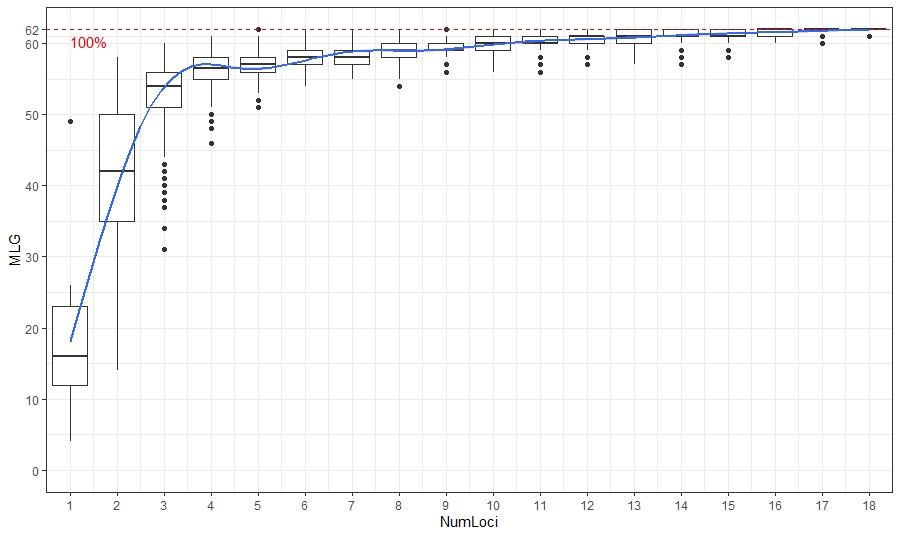

Supplement: Supplementary Figure 1 — Genotype accumulation curve with the number of loci (n-1, maximum number of loci) and the 62 discriminated MLGs confirming the suitability of the 19 SSR markers applied in the present study. [file Image_1.jpeg]
